# Esophageal achalasia detected by vomiting during induction of general anesthesia: a case report

**DOI:** 10.1186/s40981-021-00488-y

**Published:** 2021-12-10

**Authors:** Kyoko Abe, Tetsu Kimura, Yukitoshi Niiyama

**Affiliations:** 1 Department of Anesthesia and Intensive Care Medicine, Akita University Hospital, Hondo 1-1-1, Akita city, Akita 010-8543 Japan; 2 Department of Anesthesia and Intensive Care Medicine, Akita University Graduate School of Medicine, Akita, Japan

**Keywords:** Esophageal achalasia, Aspiration, Preanesthetic examination

## Abstract

**Background:**

Esophageal achalasia is a rare disease with a high risk of aspiration during anesthesia induction. Here, we describe our experience involving a case of undiagnosed esophageal achalasia with profuse vomiting during anesthesia induction.

**Case presentation:**

A 58-year-old woman was scheduled for orthopedic surgery under general anesthesia. She vomited a large amount of watery contents during anesthesia induction, and planned surgery was postponed. After recovery from anesthesia, she informed us that she usually had to drink a large amount of water to get food into her stomach and purged watery vomit every night before sleep. However, she attributed it to her constitutional problem, not to a specific disease. She was subsequently diagnosed with esophageal achalasia and underwent Heller myotomy with Dor fundoplication before her re-scheduled orthopedic surgery.

**Conclusions:**

A detailed history of dysphagia and regurgitation should be taken in preoperative examinations to prevent unexpected aspiration due to undiagnosed achalasia.

## Background

Esophageal achalasia is a rare disease (incidence, 1 in 100,000) associated with a high risk of aspiration during the induction of general anesthesia due to difficulties in passing food [1]. Here, we present a patient with undiagnosed esophageal achalasia who vomited profusely during the induction of general anesthesia.

## Case presentation

A 58-year-old woman (height 163 cm, weight 52 kg, body mass index 19.6) with a diagnosis of spinal canal stenosis was admitted to an orthopedic hospital to undergo posterior lumbar interbody fusion. Her medical history included anterior cervical fusion 12 years previously without any anesthetic complications such as allergy or bronchial asthma. No full-time anesthesiologist was on staff at the hospital, so a part-time anesthesiologist was in charge of the anesthesia.

The patient had been fasting for 15 h, from dinner the night before surgery until entering the operating room (OR). Four hours before entering the OR, an intravenous infusion was started. The patient was administered ranitidine (150 mg orally) with a small amount of water 180 min before entering the OR.

Immediately before starting the anesthesia induction, the anesthesiologist reconfirmed the patient’s medical history and whether she had any allergies or bronchial asthma. The patient did not provide any new medical information. Electrocardiography, pulse oximetry (SpO_2_), noninvasive blood pressure, and capnometry were applied. After preoxygenation with 100% oxygen for 3 min, general anesthesia was induced with fentanyl 50 μg and propofol 100 mg. After confirming the patient’s spontaneous breathing cessation, assisted ventilation was initiated with a face mask, followed by rocuronium 50 mg. Immediately after that, the patient threw up a massive amount of watery vomit. The vomit in her oral cavity was immediately suctioned, and then her trachea was intubated. A small amount of watery vomit was suctioned through the endotracheal tube. Lung compliance was low with manual ventilation, and wheezing was heard on auscultation with an obstructive pattern on capnography. Bronchospasm due to aspiration was suspected, and treatment was initiated. Under anesthesia (sevoflurane 2% in oxygen 100%), her SpO_2_ remained above 98%. Hydrocortisone 100 mg and aminophylline 250 mg were administered by drip infusion. Forty-five minutes after the event, her wheezing disappeared, and mostly normal lung compliance returned. The scheduled surgery was postponed, the patient was awakened from anesthesia with the aid of sugammadex to antagonize rocuronium, and her trachea was extubated.

After returning to the ward, a more detailed medical history was obtained from the patient. She said that she usually had to drink a large amount of water to get food into her stomach. Therefore, she made it a daily habit to purge watery vomit before bed, as it would reflux when she lay down. Although she had an upper gastrointestinal endoscopy a few years prior, the physician had not pointed out anything unusual. Since then, she believed that this was a constitutional problem as opposed to a disease. The patient had not experienced any weight loss for the past few years.

Although no clear image of pneumonia was seen on a chest X-ray at the ward, ampicillin and sulbactam were administered for prevention. The patient was discharged the next day without any medical problems. Based on an overall assessment of the episode, we believed that this patient had esophageal achalasia and advised her to visit a gastroenterologist.

**Fig. 1:**
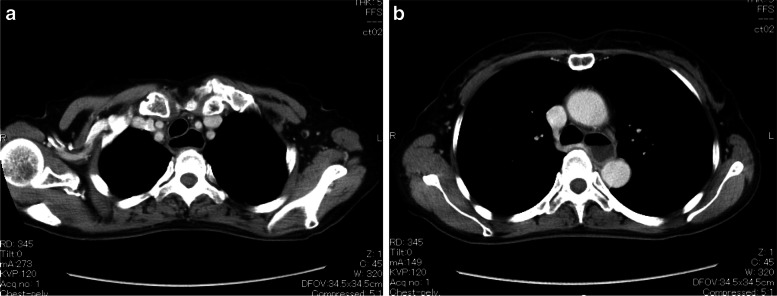
Preoperative thoracic computed tomography showing fluid retention in the dilated esophagus. **a** Upper thoracic region. **b** Middle thoracic region

**Fig. 2:**
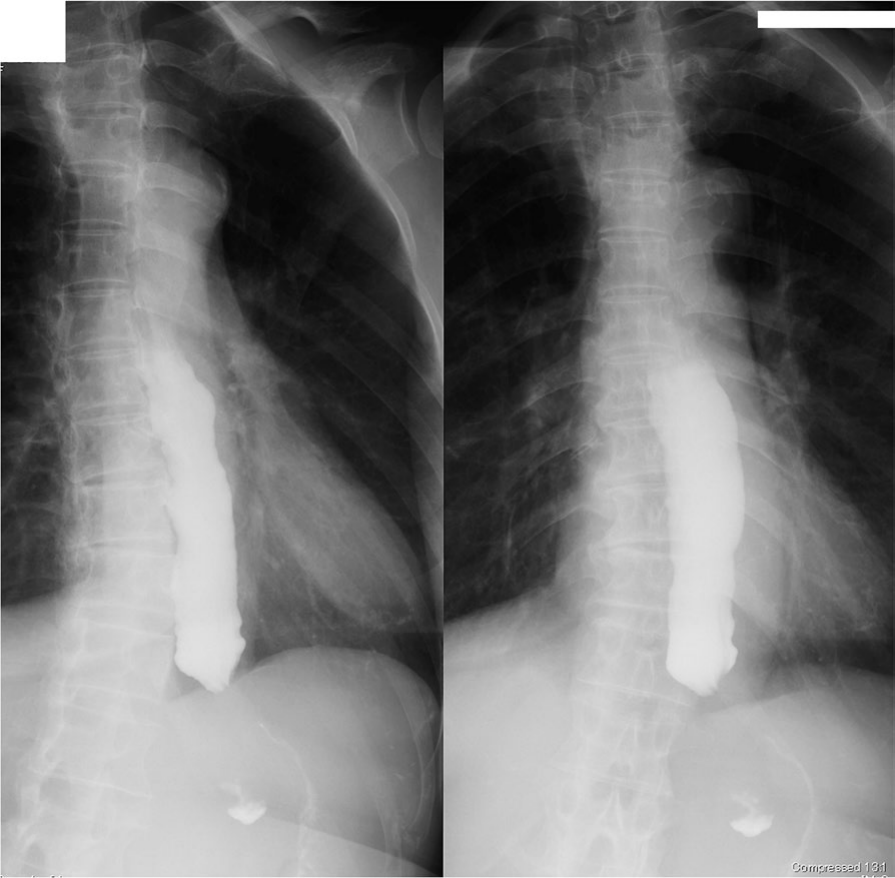
Preoperative esophagogram showed a dilated esophagus and functional narrowing of the esophagogastric junction. A mouthful of contrast medium was retained in the esophagus until the end of the imaging

At the gastroenterology department in another hospital, fluid retention in the dilated esophagus was revealed by upper endoscopy and chest computed tomography (CT) (Fig. 1). The esophagogram showed a dilated esophagus with a diameter of 32 mm and functional narrowing of the esophagogastric junction (Fig. 2). Esophageal manometry showed the disappearance of primary peristaltic waves and the occurrence of simultaneous contraction waves. Based on these results, the patient was diagnosed with esophageal achalasia and underwent laparoscopic Heller myotomy with Dor fundoplication surgery. After the surgery, her subjective symptoms, including choking with food and daily vomiting before bed, disappeared. She was discharged on the sixth day after surgery. Three months later, she underwent posterior interbody fusion of the lumbar spine without any anesthetic complications.

## Discussion

Esophageal achalasia is defined as an esophageal motility disorder of unknown etiology characterized by failure of lower esophageal sphincter (LES) relaxation and impaired peristalsis of the lower esophageal body [2]. Diagnosis is difficult because achalasia is a rare disease (incidence, 1 in 100,000 people) with nonspecific subjective symptoms [3]. The most frequent symptoms of achalasia include dysphagia of solids and liquids (> 90%), regurgitation of undigested food (76–91%), respiratory complications such as nocturnal cough (30%) and aspiration (8%), chest pains (25–64%), heartburn (18–52%), and weight loss (35–91%) [4]. Oral reflux in patients with achalasia does not originate from the stomach and thus does not contain acidic contents. Furthermore, if the volume of the oral reflux is mild, the patient may be unaware of the reflux.

We assume two reasons for the aspiration during the first scheduled orthopedic surgery in this case. First, the patient was not aware of her rare disease. She had unusual habits such as washing food into her stomach with large amounts of water and purging watery vomit every night before bed. Nonetheless, she did not consider it unusual because her gastroenterologist told her that nothing was wrong. The diagnostic features of esophageal achalasia on upper gastrointestinal endoscopy include (1) dilatation of the esophageal lumen, (2) abnormal retention of food and fluid in the esophagus, (3) whitening and thickening of the esophageal mucosal surface, (4) functional narrowing of the esophagogastric junction, and (5) abnormal contraction waves of the esophagus [2]. In this case, preoperative endoscopy before Heller and Dor surgery showed fluid retention in the esophagus and functional narrowing of the esophagogastric junction, but the dilation of the esophageal lumen was mild. As a result, the gastroenterologist at the time was unable to detect achalasia, leading to her misconception that it was not a disease.

Second, we could not obtain information regarding dysphagia and food regurgitation in the preoperative examination. Our routine preoperative examination for orthopedic patients without gastrointestinal complications includes a detailed medical history, allergies, and asthma, but not dysphagia or food regurgitation. In patients with esophageal achalasia, 37% have solid residue, and 14.8% retain water, even after fasting for 24 h before surgery [5]. Fortunately, because this patient had a habit of vomiting every night before bed, and the vomit did not contain any solids, she did not develop severe pneumonia.

**Fig. 3:**
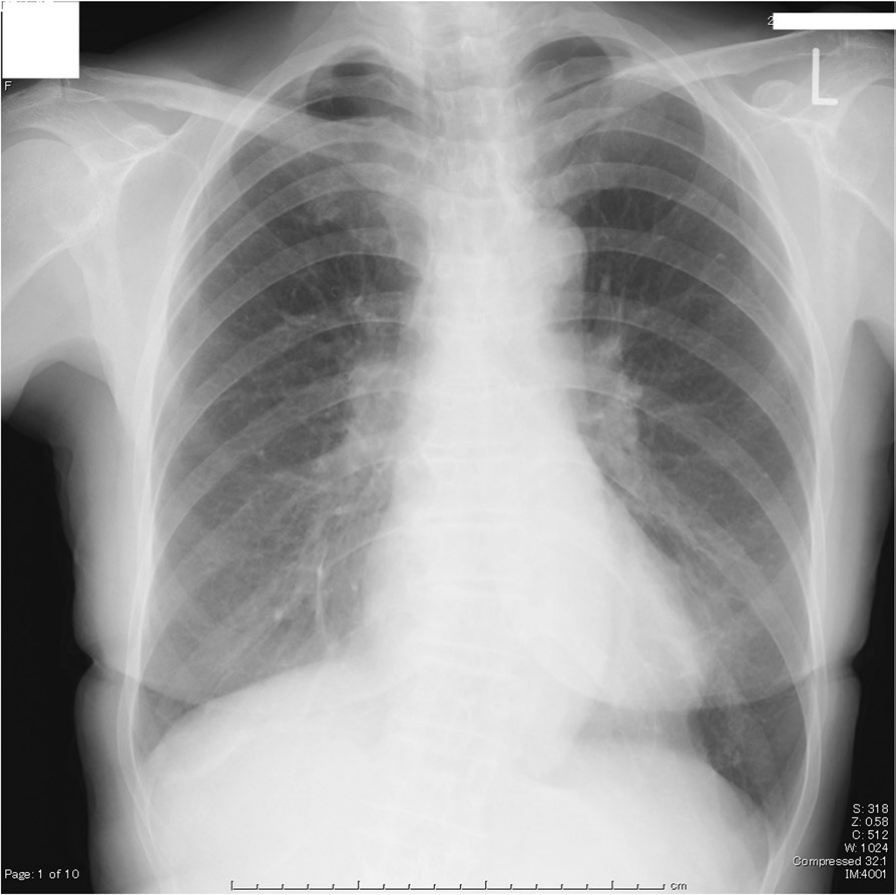
Preoperative chest X-ray did not show any abnormalities

Achalasia is a rare disease, and only a few cases have been detected by aspiration during the induction of anesthesia [1, 6, 7]. Since achalasia is a chronic benign disease, patients may recognize it as a constitutional problem, as in this case. A detailed history of dysphagia and regurgitation should be taken from all patients during preoperative examinations to avoid aspiration risk, even if achalasia is not suspected. Pillow stains with drool while sleeping suggests the existence of achalasia. Although chest X-rays appear normal in the early phase of achalasia, the dilated esophagus creates new interfaces with the lung as the disease progresses, which makes achalasia-specific findings, including convex opacity overlapping the right mediastinum, air-fluid levels in the thorax esophagus, and small or absent gastric bubbles [8]. In this case, the esophageal dilatation was so mild that no suspicious contour was seen on the chest X-ray (Fig. 3). However, it should be worthwhile for physicians to get into the habit of looking for achalasia-specific findings on routine X-ray readings to detect undiagnosed achalasia. Furthermore, we would like to emphasize that achalasia should not be ruled out even if there are no findings specific to achalasia, especially in patients with symptoms such as dysphagia or regurgitation. If a chest CT is taken for the preoperative evaluation, physicians should also note the dilated image of the esophagus.

## Conclusions

Although esophageal achalasia is a rare disease, it is associated with a high risk of aspiration pneumonia if general anesthesia is induced without caution. A detailed history of dysphagia and regurgitation of solids and liquids in the preoperative examination is essential and practical for detecting the existence of achalasia and preventing unexpected aspiration due to undiagnosed achalasia. Additionally, getting into the habit of checking for achalasia-specific findings on chest X-rays and chest CT may reduce the occurrence of unexpected aspiration.
